# New Pieces in the Puzzle of uPAR Role in Cell Migration Mechanisms

**DOI:** 10.3390/cells9122531

**Published:** 2020-11-24

**Authors:** Anna Gorrasi, Anna Maria Petrone, Anna Li Santi, Mariaevelina Alfieri, Nunzia Montuori, Pia Ragno

**Affiliations:** 1 Department of Chemistry and Biology, University of Salerno, 84084 Fisciano (Salerno), Italy; angorrasi@gmail.com (A.G.); annamariapetrone1988@gmail.com (A.M.P.); alisanti@unisa.it (A.L.S.); malfieri@unisa.it (M.A.); 2 Department of Translational Medical Sciences, “Federico II” University, 80138 Naples, Italy; nmontuor@unina.it

**Keywords:** urokinase receptor, uPAR, fMLF receptors, FPR1, cell migration

## Abstract

The urokinase (uPA) receptor (uPAR) plays a key role in cell migration. We previously showed that uPAR-negative HEK-293 cells efficiently migrate toward serum but, after uPAR ectopic expression, migrate only in a uPAR-dependent manner. In fact, migration of uPAR-transfected HEK-293 (uPAR-293) cells is impaired by anti-uPAR antibodies, without recovery of the uPAR-independent migration mechanisms formerly active. Prostate carcinoma PC3 cells, which express high endogenous uPAR levels, migrated only through a uPAR-dependent mechanism; in fact, the silencing of uPAR expression inhibited their migration. We hypothesize a crucial role of the uPAR glycosyl-phosphatidyl-inositol (GPI) tail, which promotes uPAR partitioning to lipid rafts, in uPAR-controlled cell migration. Here, we show that removal of the uPAR GPI-tail, or lipid rafts disruption by methyl-beta-cyclodextrin impairs migration of PC3 cells, incapable of uPAR-independent migration, whereas it restores uPAR-independent migration in uPAR-293 cells. We then show that, in PC3 cells, both uPAR signaling partners, β1 integrins and receptors for formylated peptides (FPRs), partly associate with lipid rafts. Inhibition of their interaction with uPAR impairs this association and impairs cell migration. Interestingly, blocking uPAR association with FPRs also impairs β1 integrin partitioning to lipid rafts, whereas blocking its association with β1 integrins has no effect on FPRs partitioning. On these bases, we propose that uPAR controls cell migration by connecting β1 integrins and FPRs and, through its GPI tail, by driving them into lipid rafts, thus promoting pro-migratory signals. uPAR-mediated partitioning of integrins to lipid rafts is strictly dependent on uPAR association with FPRs.

## 1. Introduction

The urokinase receptor (uPAR) is a three-domain receptor anchored to the cell surface through a glycosyl-phosphatidyl-inositol (GPI) tail [1]. uPAR resolved structure showed that the three domains form a cavity hosting its ligand, the urokinase-type plasminogen activator (uPA), which binds sites mainly located in the N-terminal Domain I (DI); the large outer surface of the receptor is available to bind additional ligands [2]. In fact, uPAR is also a non integrinic receptor for vitronectin (VN), an extracellular matrix (ECM) component, and it associates with various cell surface molecules, in particular with integrinic adhesion molecules [3] and with the G-protein coupled receptors for formylated peptides as fMLF (FPRs) [4].

Since its identification, uPAR has been considered a key molecule in the regulation of the pericellular proteolysis needed for migration of cells through the ECM. In fact, it concentrates uPA and, thus, plasmin generation, on the cell surface, allowing localized degradation of almost all components of the ECM. However, uPAR-mediated cell migration towards uPA and vitronectin, independent of uPA protease activity, has since been largely demonstrated. Furthermore, uPA-induced cell migration was shown to require FPR expression, and, conversely, fMLF-induced cell migration requires uPAR expression [1]. Thus, uPAR, despite being anchored to the cell surface through a GPI-tail, is able to activate intracellular signaling pathways promoting cell migration; integrins and FPRs, interacting with uPAR, act as signaling partners mediating signals transduction across the cytoplasmic membrane [5].

Accordingly, uPAR plays a direct role in physiological and pathological processes that require cell migration [6,7].

We previously demonstrated that uPAR takes control of mechanisms of directional cell migration by interacting with its signaling partners, i.e., integrins and FPRs [8,9]. We showed that uPAR-negative HEK-293 cells were able to migrate efficiently towards serum, thus through a uPAR-independent mechanism, but, after uPAR ectopic expression, they migrated only in a uPAR-dependent manner. In fact, a polyclonal antibody against uPAR, inhibition of uPAR interaction with integrins, or desensitization of FPRs expressed in HEK-293 cells, impaired directional migration of uPAR-transfected 293 (uPAR-293) cells, without restoring their ability to migrate in the absence of uPAR expression [9]. Thus, uPAR expression is not required for directional cell migration of uPAR-negative HEK-293 cells, but, once uPAR is expressed on their surface, it takes control of migration mechanisms (uPAR-dependent mechanism). This regulatory capability does not only involve its signaling partners, since blocking these interactions does not restore migration. We also showed that silencing uPAR expression in prostate carcinoma PC3 cells, which constitutively express high amounts of uPAR [10], impaired their migration towards serum, suggesting that these cells are able to migrate only using uPAR-controlled mechanisms [9].

Since neutralization of uPAR interactions with its signaling partners failed to restore uPAR-independent cell migration mechanism in uPAR-293 cells, we propose a crucial role for the GPI tail of uPAR in the control of directional cell migration. In fact, the GPI anchor allows several cell-surface molecules, including uPAR, to associate with specialized microdomains of the plasma membrane, the lipid rafts, which play a crucial role in connecting intracellular signaling mediators to surface receptors [11,12].

In this study, we aimed to elucidate better the importance and the role of the GPI anchor in uPAR-controlled cell migration towards serum. We focused our studies on PC3 cells, which require uPAR expression for their migration and compared them with uPAR-293 cells. In fact, uPAR-293 cells derive from cells able to migrate even in the absence of uPAR and, therefore, allow us to investigate the selective effects of GPI-tail removal or lipid rafts disruption on uPAR-controlled or on uPAR-independent mechanisms of migration. We first determined whether uPAR-dependent PC3 cell migration follows the same mechanisms as uPAR-293 cell migration. Then, we focused on the mechanisms underlying the role that uPAR GPI-tail and uPAR association with lipid rafts play in cell migration.

## 2. Materials and Methods

### 2.1. Reagents

The anti-uPAR monoclonal antibody (mAb), clone R4 (cat. MON R-4-02), used for Western blot analysis, was from Thermo Fisher Scientific (Rockford, IL, USA). The anti-uPAR polyclonal antibody (sc-10815), used in cell migration assays, the mAb against β1 integrins (sc-13590), the mAb anti-caveolin-1 (sc-53564), the mAb against FPR2 (sc-57141) and the anti-FPR3 polyclonal antibody (sc-66900) were from Santa Cruz Biotechnology (Santa Cruz, CA, USA). The anti-FPR1 polyclonal antibody (ab113531) was purchased from Abcam (Cambridge, MA, USA). The mAb anti-tubulin antibody (bsm-50179M) was from Bioss antibodies (Woburn, MA, USA). Anti-GAPDH monoclonal (cat. G041) and polyclonal (cat. Y058203) antibodies were purchased from abm (Vancouver, BC, Canada). The rabbit antibody recognizing the SRSRY sequence of uPAR was developed by PRIMM (Milan, Italy) by using the uPAR_84–95_ peptide (corresponding to uPAR residues 84–95, which include the SRSRY sequence) assembled onto a branching lysine core [8].

The protease inhibitor cocktail, Methyl-beta-cyclodextrin and Collagen IV were from SIGMA (St. Louis, MO, USA). Phosphatidylinositol-specific phospholipase C (cat. 1143077), from B. cereus, was from Boehringer Mannheim (Mannheim, Germany). Calbiochem Rac1 (CAS 1177865-17-6) and Rho kinase (H1152P) inhibitors were purchased from Millipore (Burlington, MA, USA). Invitrogen Lipofectamine 2000, horseradish peroxidase-conjugated anti-mouse and anti-rabbit IgG were from Thermo Fisher Scientific (Rockford, IL, USA). ECL detection kit was from Amersham International (Amersham, UK) and the Polyvinylidene fluoride (PVDF) membrane from Millipore (Burlington, MA, USA). The chemotaxis polyvinylpyrrolidone-free (PVPF) filters were from Whatman Int. (Kent, UK). The W (WKYMVm) peptide, the P25 (AESTYHHLSLGYMYTLN) peptide and its scrambled version (NYHYLESSMTALYTLGH) [13] were synthesized by PRIMM (Milan, Italy).

### 2.2. Cell Culture

Prostate carcinoma (PC3) cells were grown in DMEM supplemented with 10% fetal bovine serum (FBS). uPAR-293 cells are HEK-293 stably transfected with the entire coding region of uPAR cDNA cloned in the EcoRI site of the pcDNA3 vector [14]; stably transfected cells were grown in DMEM supplemented with 10% FBS and 0.5 mg/mL Geneticin.

### 2.3. Cell Migration Assay

Cell migration assays were performed in Boyden chambers using 8 µm pore size PVPF polycarbonate filters coated with 50 µg/mL collagen, as previously described [9]. Briefly, 2 × 10^5^ uPAR-293 cells or 1 × 10^5^ PC3 cells were loaded in the upper chamber in serum-free medium; 10% FBS-DMEM was added in the lower chamber as chemoattractant. Cells were incubated for 4 h (uPAR-293 cells) and 2 h (PC3 cells) at 37 °C, 5% CO_2_. Then, the cells on the lower surface of the filter were fixed in ethanol, stained with hematoxylin, and counted at 200× magnification (10 random fields/filter). When indicated, cells were pre-incubated with 5 µg/mL polyclonal antibodies directed to full-length uPAR or uPAR_84–95_ region, or with 50 µM P25 peptide for 1 h at room temperature, or with 50 µM C6 compound for 30 min at 37 °C, 5% CO_2_, or with 10 µM inhibitor of Rho signaling or 50 µM inhibitor of Rac signaling for 30 min at 37 °C, or with 5 nM W peptide for 1 h at 37 °C, 5% CO_2_.

### 2.4. TM-uPAR Construct

A chimeric cDNA construct encoding the extracellular domains of uPAR (amino acids 1–281) and the transmembrane and cytoplasmic domains of the α subunit of the Interleukin-2 receptor (IL-2R α, amino acids 218-251) was prepared (TM-uPAR).

The cDNA for uPAR 1-281 was obtained by PCR amplification of the uPAR cDNA [14] with following primers: CGG GGT ACC ATG GGT CAC CCG CCG CTG CTG CCG (forward); CCG GAA TTC GTT CAT GCT GAA GGC GTC AC (reverse). The resulting KpnI-EcoRI fragment was cloned in the pcDNA3 plasmid (termed uPAR1-281 pcDNA3). The cDNA for IL-2R α fragment was prepared by retrotranscription of total RNA isolated from U937 cells and PCR amplification with the following primers: CCG GAA TTC ACA GAT TTT CAA ATA CAG ACA GA (forward); CCG CTC GAG CTA GAT CAG CAG GAA AAC AC (reverse). The obtained EcoRI-Xho fragment was cloned in the uPAR1-281 pcDNA3. The nucleotide sequence and the orientation of inserted fragments were confirmed by DNA sequencing.

### 2.5. Transfections

uPAR-293 cells are HEK-293 stably transfected with full-length uPAR cDNA cloned in the EcoRI site of the pcDNA3 vector [14]. GPI-uPAR-293 and TM-uPAR 293 cells are HEK-293 transiently transfected with full-length uPAR cDNA [14] and TM-uPAR cDNA, respectively. In both cases, 2.5 × 10^6^ cells, plated in 60 mm dishes, were transfected with 9 μg of DNA and 22.5 μL of LipofectAMINE 2000 (Invitrogen, Paisley, UK) in serum-free DMEM for 5 h at 37 °C (5% CO_2_). Cells were harvested 48 h after transfection and used for migration assays or lysed and analyzed by Western blot analysis.

### 2.6. PI-PLC and MCD Treatment

Cells were incubated with or without PI-PLC (1 U/mL) for 1 h at 37 °C, 5% CO_2_. Washed cells were subjected to migration assays; remaining cells were lysed in 1% Triton X-100 containing a cocktail of protease inhibitors, for Western blot analysis.

Methyl-beta-cyclodextrin (MCD) treatment included an incubation in 10 mM MCD for 15 min (uPAR-293 cells) o 30 min (PC3 cells) at 37 °C. Washed cells were then subjected to migration assays or lysed for lipid rafts analysis.

### 2.7. Lipid Rafts Analysis

Association of indicated proteins with lipid rafts was evaluated by density gradient centrifugation. Cells were lysed in lysis buffer (20 mM MOPS, 0.15 M NaCl, 1% Triton-X 100 and protease inhibitors) for 30 min on ice. The protein content was measured by a colorimetric assay (Bio-Rad, Munchen, Germany) and 1 mg of protein was diluted to 0.9 mL, mixed with 0.9 mL of 80% sucrose in MOPS and placed at the bottom of a centrifuge tube. Samples were overlaid with 1.8 mL of 30% and 0.9 mL of 5% sucrose in MOPS. The gradient was then centrifuged at 32,500 rpm for 24 h at 4 °C using a BECKMAN SW50.1 rotor. After centrifugation 0.45 mL fractions were collected from the top to the bottom of the gradient; the last five fractions (bottom) were considered non-raft fractions.

Equal volumes (45 µL) of each fraction were analyzed by Western blot analysis with specific antibodies.

### 2.8. Western Blot Analysis

Cells were lysed in 1% Triton X-100 and protease inhibitors; the protein content was measured by a colorimetric assay (Bio-Rad). Cell lysates were electrophoresed in SDS-PAGE, transferred onto a PVDF membrane, blocked with 5% milk and probed with primary antibodies. Washed membranes were incubated with horseradish peroxidase-conjugated secondary antibodies and bands detected by ECL. For loading control, membranes were re-probed with anti-tubulin or anti-GAPDH primary antibody.

### 2.9. Statistical Analysis

Differences between each group of values and its control group were evaluated by the Student’s *t*-test using PRISM software (GraphPad, San Diego, CA, USA). *p* < 0.05 was considered statistically significant.

## 3. Results

### 3.1. uPAR Interactions with FPRs and β1 Integrins Are Involved in Migration of Prostate Carcinoma PC3 Cells

We first determined whether migration of PC3 cells, which express high levels of endogenous uPAR, is regulated by same mechanisms regulating the migration of uPAR-transfected 293 cells.

In particular, we assessed whether uPAR interactions with the β1 integrins expressed in PC3 cells [15], and with FPRs play a role in PC3 cell migration as previously shown in uPAR-293 cell migration [9].

**Figure 1:**
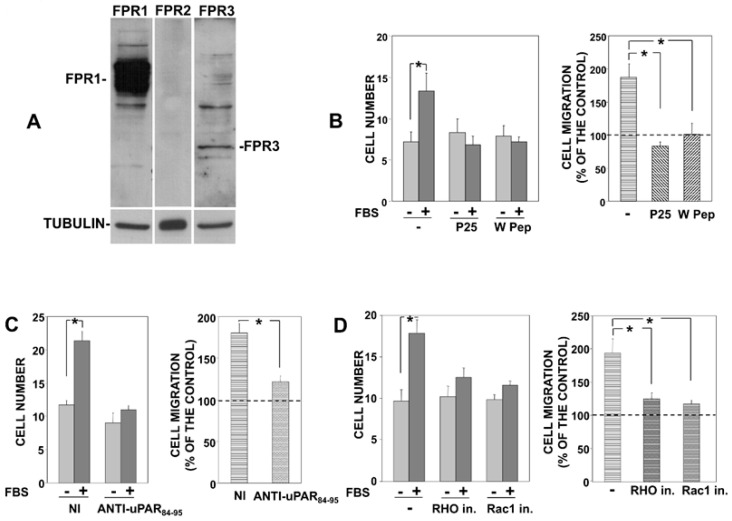
uPAR interactions with FPR1 and β1 integrins are involved in migration of prostate carcinoma PC3 cells. PC3 cells were lysed and 50 μg of cell extract was analyzed by Western blot with indicated antibodies; membranes were reprobed with an anti-tubulin mouse antibody for loading control (**A**). PC3 cells were pre-incubated with diluent (−) or 5 nM W Peptide (W Pep) or 50 µM P25 (**B**) or with 5 µg/mL nonimmune Ig (NI) or an antibody directed to 84–95 uPAR residues (**C**) or with diluent (−) or 10 µM Rho kinase inhibitor or 20 µM Rac1 inhibitor (**D**). Cells were then plated in Boyden chambers and allowed to migrate towards 10% fetal bovine serum (FBS). Migrated cells were fixed, stained with hematoxylin, and counted ((**B**–**D**) left panels). Results of migration assays are also expressed as percentage of cells migrated towards serum over the cells migrated without serum; 100% values represent cell migration in the absence of chemoattractants ((**B**–**D**) right panels). The values are the mean + SD of three experiments performed in triplicate. (*) *p* < 0.05, as determined by the Student’s *t*-test.

Firstly, we evaluated the expression of FPRs in PC3 cells, showing that FPR1 is the main FPR expressed in PC3 cells (Figure 1A), as previously shown also in uPAR-293 cells [9].

Then, we impaired the association of uPAR with integrins or FPR1 in PC3 cells and evaluated their migration. PC3 cells were treated with the P25 peptide, which competes with integrins for association to uPAR [13], or with the W peptide (W Pep), which is a ligand of FPRs, able to desensitize them and to induce their internalization [16]. Treated cells were then allowed to migrate towards serum, a generic chemoattractant. Results showed that serum-induced PC3 cell migration was significantly inhibited by both peptides (Figure 1B), indicating that uPAR interactions with integrins and FPRs are crucial for PC3 cell migration, as previously shown for uPAR-293 cell migration.

The central role of FPRs was further confirmed by a polyclonal antibody directed against the uPAR region (aminoacidic residues 84-95) involved in its association to FPRs. In fact, this anti-uPAR antibody significantly inhibited PC3 cell migration towards serum (Figure 1C). Neither the peptides nor the specific antibody significantly influenced basal migration of PC3 cells, that is, cell migration in the absence of chemoattractants.

Finally, we also assessed whether signaling mediators involved in uPAR signaling play a role in PC3 cell migration, as previously shown in uPAR-293 cell migration [9]. Serum-induced PC3 cell migration was inhibited by inhibitors of Rac1 and of the Rho-associated kinase, indicating Rac1 and Rho involvement in uPAR-controlled migration of PC3 cells (Figure 1D), as previously shown for uPAR-293 cells [9].

Together, these results demonstrate that directional migration of PC3 cells, which express endogenous uPAR [10], involves the same interactors and signaling mediators previously reported to be involved in the directional migration of uPAR-293 cells.

### 3.2. Removal of uPAR GPI-Anchor Impairs the uPAR-Controlled Mechanism of Cell Migration

We then focused on the role of the uPAR GPI-tail in migration of both PC3 and uPAR-293 cells.

**Figure 2:**
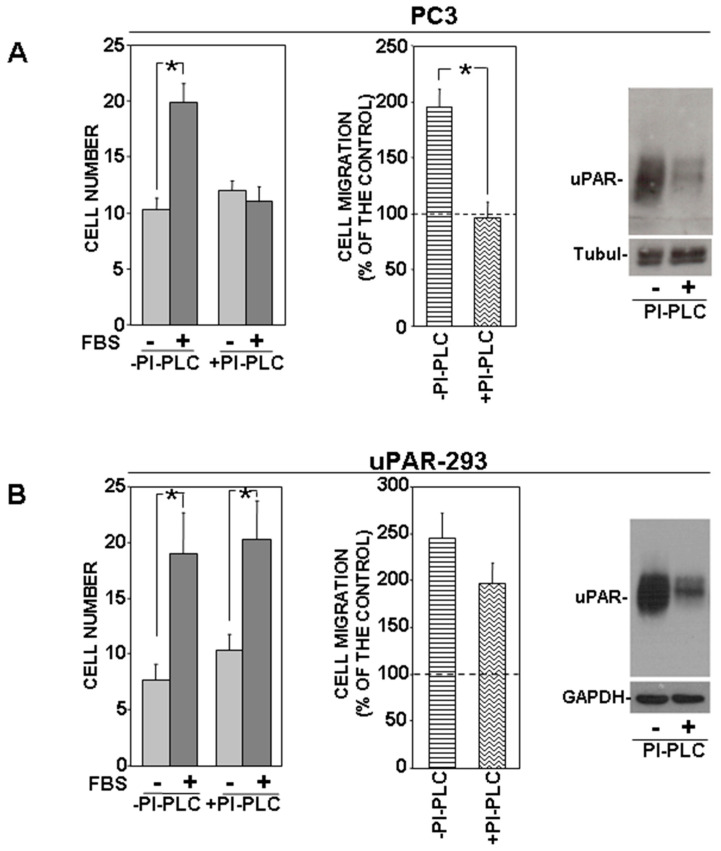
Removal of uPAR GPI-anchor impairs uPAR-controlled mechanism of cell migration. PC3 (**A**) and uPAR-293 (**B**) cells were treated with 1 U/mL of phosphatidylinositol-specific phospholipase C (PI-PLC) or with diluent. Cells were then plated in Boyden chambers and allowed to migrate towards 10% fetal bovine serum (FBS). Migrated cells were fixed, stained with hematoxylin, and counted (left graphs). Results of migration assays are also expressed as percentage of cells migrated towards serum over the cells migrated without serum; 100% values represent cell migration in the absence of chemoattractants (right graphs). The values are the mean ± SD of three experiments performed in triplicate. (*) *p* ≤ 0.05, as determined by the Student’s *t*-test. PC3 and uPAR-293 cells treated with or without PI-PLC were also lysed and analyzed by Western blot with an anti-uPAR monoclonal antibody to assess PI-PLC effect; membranes were then reprobed with anti-tubulin or anti-GAPDH rabbit antibodies for loading control ((**A**,**B**), right panels).

PC3 cells were treated with or without phosphatidylinositol-specific phospholipase C (PI-PLC) and allowed to migrate towards serum. PI-PLC treatment completely abrogated directional migration of PC3 cells (Figure 2A). PI-PLC activity on the cell-surface uPAR was assessed by Western blot analysis of PI-PLC treated or untreated cells (Figure 2A, right panel).

**Figure 3:**
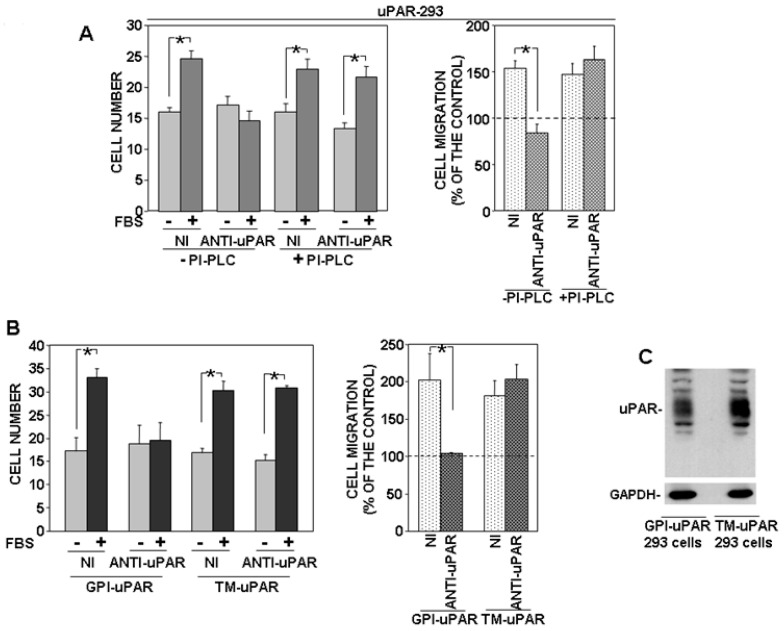
GPI-tail of uPAR mediates its control of cell migration mechanisms. (**A**) uPAR-293 cells were incubated with 1 U/mL of phosphatidylinositol-specific phospholipase C (PI-PLC) or with diluent. Cells were then treated with 5 μg/mL polyclonal anti-uPAR antibody or nonimmune Ig (NI) for 1 h and plated in Boyden chambers to migrate towards 10% fetal bovine serum (FBS). Migrated cells were fixed, stained with hematoxylin, and counted (left graph). Results of migration assays are also expressed as percentage of cells migrated towards serum over the cells migrated without serum; 100% values represent cell migration in the absence of chemoattractants (right graph). The values are the mean ± SD of three experiments performed in triplicate. (*) *p* ≤ 0.05, as determined by the Student’s *t*-test. (**B**) HEK-293 cells were transiently transfected with cDNAs of transmembrane chimeric uPAR (TM-uPAR) or full length uPAR (GPI-uPAR) as a control. Transfected cells were incubated with 5 μg/mL polyclonal anti-uPAR antibody or nonimmune Ig (NI) for 1 h and then plated in Boyden chambers and allowed to migrate towards 10% FBS. Migrated cells were fixed, stained with hematoxylin, and counted (left graph). Results of migration assays are also expressed as percentage of cells migrated towards serum over the cells migrated without serum; 100% values represent cell migration in the absence of chemoattractants (right graph). The values are the mean ± SD of three experiments performed in triplicate. (*) *p* ≤ 0.05, as determined by the Student’s *t*-test. (**C**): GPI-uPAR and TM-uPAR expression in transiently transfected cells was assessed by Western blot analysis with the same anti-uPAR polyclonal antibody used in cell migration assays; membranes were then reprobed with an anti-GAPDH mouse antibody for loading control.

By contrast, PI-PLC treatment did not influence significantly uPAR-293 cell migration, despite a comparable effect of PI-PLC (Figure 2B). The same cell migration assays were then performed in the presence of an anti-uPAR polyclonal antibody, in order to investigate whether migration of PI-PLC-treated uPAR-293 cells was still controlled by the residual uPAR expression or whether it was uPAR-independent. Results showed that the anti-uPAR antibody impaired migration of PI-PLC untreated cells, as expected, whereas it did not exert any effect on PI-PLC treated cells (Figure 3A).

To better elucidate GPI-tail role in uPAR-controlled mechanisms of migration, we prepared a construct encoding a chimeric receptor (transmembrane uPAR: TM-uPAR) composed of the extracellular domains of uPAR (amino acids 1-281) and the transmembrane and cytoplasmic domains of the *α* chain of the IL-2 receptor (IL-2R*α*) [13]. HEK-293 cells were transiently transfected with TM-uPAR cDNA and full length uPAR (GPI-uPAR) cDNA, as a control. Transfected cells were allowed to migrate towards serum, in the presence of nonimmune Ig or the anti-uPAR polyclonal antibody (Figure 3B). Both GPI-uPAR 293 cells and TM-uPAR 293 cells efficiently migrated; however, again, the anti-uPAR antibody impaired migration of GPI-uPAR 293 cells, as expected, whereas it did not exert any effect on migration of TM-uPAR 293 cells, indicating that in the latter cells, uPAR, deprived of its GPI-tail, loses its capability to control migration mechanisms. uPAR expression in transfected cells was assessed by Western blot using the same anti-uPAR polyclonal antibody used in migration assays. Western blot analysis showed that cells transiently transfected with GPI-uPAR or TM-uPAR cDNAs expressed similar levels of uPAR (Figure 3C).

Together, these results indicate that uPAR, when expressed, may take control of cell migration mostly because of its GPI-tail.

### 3.3. Disruption of Lipid Rafts Impairs uPAR-Controlled Cell Migration

**Figure 4:**
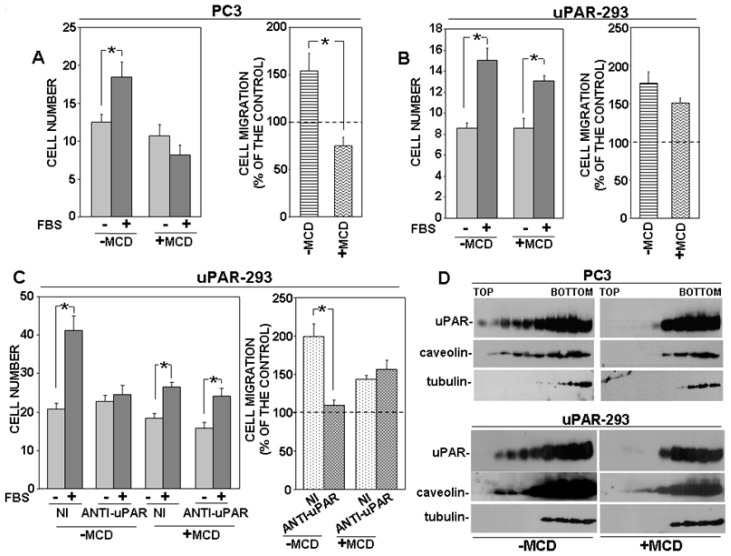
Disruption of lipid rafts impairs uPAR-controlled cell migration. PC3 (**A**) or uPAR-293 (**B**) cells were treated with or without 10 mM methyl-beta-cyclodextrin (MCD). Cells were then plated in Boyden chambers and allowed to migrate towards 10% fetal bovine serum (FBS). MCD treated or untreated uPAR-293 cells were also incubated with 5 μg/mL polyclonal anti-uPAR antibody or nonimmune Ig (NI) before migration (**C**). Migrated cells were fixed, stained with hematoxylin, and counted (left panels). Results of migration assays are also expressed as percentage of cells migrated towards serum over the cells migrated without serum; 100% values represent cell migration in the absence of chemoattractants (right panels). The values are the mean + SD of three experiments performed in triplicate. (*) *p* < 0.05, as determined by the Student’s *t*-test. PC3 and uPAR-293 cells treated with or without MCD were also lysed in buffer containing 1% Triton X-100. Cell lysates (1 mg) were loaded onto a sucrose density gradient and subjected to ultracentrifugation. Then, 4.5 mL were harvested as 0.45 mL fractions. Equal volumes of each fraction were analyzed by Western blot with an anti-uPAR monoclonal antibody, or with anti-tubulin or anti-caveolin antibodies, as controls of non-raft associated proteins or raft associated proteins, respectively (**D**).

GPI proteins, including uPAR, may associate with lipid rafts, cholesterol-rich microdomains of the cell membrane [17]. Thus, we investigated whether disruption of lipid rafts may induce the same effects of PI-PLC removal on migration of uPAR-expressing cells. Both PC3 and uPAR-293 cells were treated with MCD, which depletes lipid rafts of cholesterol, inducing their disruption; then, cells were assayed for migration towards serum. Cell behavior was similar to that observed after PI-PLC treatment or with TM-uPAR 293 cells. In fact, MCD abrogated migration of PC3 cells, whereas it did not exert any significant effect on migration of uPAR-293 cells (Figure 4A,B, respectively). Again, the anti-uPAR antibody impaired migration of untreated uPAR-293 cells, whereas it did not exert any significant effect on migration of MCD-treated uPAR-293 cells, demonstrating that, in the latter case, cell migration was regulated by a uPAR-independent mechanism (Figure 4C).

The effect of MCD on the partition of uPAR to lipid rafts was assessed by loading lysates of MCD-treated or untreated cells on sucrose gradients, in which, after ultracentrifugation, proteins associated with lipid rafts tend to float to the top fractions of the gradient. Fractions harvested after ultracentrifugation were analyzed by Western blot with specific antibodies. Results showed that uPAR associated with lipid rafts in MCD-untreated cells, as expected, in both PC3 and uPAR-293 cells; caveolin was used as a control of lipid raft-associated protein and tubulin as marker for non-raft fractions (Figure 4D, left panels). In MCD-treated cells, uPAR and caveolin were only recovered in non-raft fractions, together with tubulin, confirming the efficacy of MCD treatment (Figure 4D, right panels).

These results strongly suggest that the role of the uPAR GPI-tail in the uPAR-controlled migration mechanism is likely related to its capability to drive the receptor to lipid rafts.

### 3.4. uPAR Drives Its Signaling Partners to Lipid Rafts

We then investigated whether uPAR also drives its signaling partners, β1 integrins and FPR1, to the lipid rafts. Interaction of β1 integrins and FPR1 with uPAR is required for uPAR-controlled cell migration. We focused our studies on PC3 cells, in which only this type of migration occurs.

**Figure 5:**
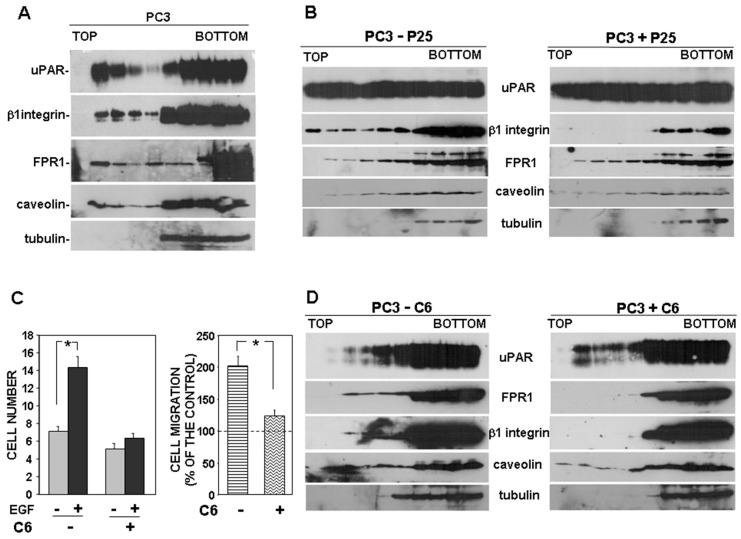
uPAR drives its signaling partners to lipid rafts. Cultured PC3 cells (**A**) or PC3 cells treated with 50 μM P25 or with its scrambled version (−) (**B**) were lysed in buffer containing 1% Triton X-100. Cell lysates were loaded onto a sucrose density gradient and subjected to ultracentrifugation. Then, 4.5 mL were harvested as 0.45 mL fractions; 45 μL of each fraction was analyzed by Western blot with the indicated antibodies; tubulin and caveolin were used as controls of non-raft associated proteins or raft associated proteins, respectively. (**C**) PC3 cells were pre-incubated with or without 50 μM C6 compound, then plated in Boyden chambers and allowed to migrate toward 100 ng/mL EGF Epidermal Growth Factor (EGF). Migrated cells were fixed, stained with hematoxylin, and counted (left panel). Results of migration assays are also expressed as percentage of cells migrated towards serum over the cells migrated without serum; 100% values represent cell migration in the absence of chemoattractants (right panels). The values are the mean + SD of three experiments performed in triplicate. (*) *p* < 0.05, as determined by the Student’s *t*-test. C6 treated or untreated cells were also lysed in buffer containing 1% Triton X-100, loaded onto a sucrose density gradient and subjected to ultracentrifugation. Then, 45 μL of each gradient fraction was analyzed by Western blot with the indicated antibodies; tubulin and caveolin were used as control of non-raft associated proteins and raft associated proteins, respectively (**D**).

First, we investigated where uPAR signaling partners partitioned in basic conditions. Western blot analysis of gradient fractions showed that uPAR, as expected, partly associated with lipid raft fractions, as well as both β1 integrins and FPR1; caveolin and tubulin were used as controls (Figure 5A).

Then, we investigated the possible role of uPAR in the observed partitioning of β1 integrins to lipid rafts, by using the P25 peptide, which inhibits uPAR-β1 integrins interaction [13]. PC3 cells were treated with the P25 peptide or its scrambled version; then, cell lysates were analyzed by sucrose gradient and Western blot analysis with specific antibodies. Results showed that, abrogating the uPAR–integrin interaction impaired β1 integrin partition to lipid rafts, whereas it did not affect FPR1 association with lipid rafts (Figure 5B).

Then, we investigated the possible uPAR role in the observed partition of FPR1 to lipid rafts. We used the C6 compound to impair uPAR-FPR1 interaction. C6 is a small molecule that specifically targets the uPAR region interacting with FPR1, thus preventing uPAR co-immunoprecipitation with FPR1 [18]. It has been previously shown that C6 compound impaired PC3 cell invasion of MATRIGEL, thus we first assessed whether it was also able to inhibit PC3 cell directional migration. Since the uPAR binding site for FPR1 includes two aminoacidic residues that are also critical for the binding to VN, we performed migration assays towards the epidermal growth factor (EGF) instead of serum, which may contain VN. We confirmed the inhibitory effect of C6 on PC3 cell migration (Figure 5C). Then, we investigated C6 effect on FPR1 and β1 integrins partitioning to lipid rafts. PC3 cells were treated with or without C6, lysed, loaded on sucrose gradients and subjected to ultracentrifugation. Western blot analysis with specific antibodies showed that abrogation of uPAR-FPR1 interaction impairs FPR1 partitioning to lipid rafts. Interestingly, impairment of the uPAR-FPR1 interaction also affects β1 integrins association with lipid rafts (Figure 5D).

Together, these results suggest that uPAR drives its signaling partners to lipid rafts, where signaling activity is more efficient, and that this function is FPR1-dependent.

## 4. Discussion

uPAR association with various integrin families and with FPRs has been widely demonstrated. Integrins and FPRs act as uPAR signaling partners, since uPAR is a GPI cell-anchored protein, and is thus unable to transduce signals across cell surface [5]. On the other hand, uPAR also seems to regulate the activity of its signaling partners [3]. uPAR can associate with various proteins, both in lipid rafts and in non-raft domains of the plasma membrane of human neutrophils [19]. uPAR partitioning to lipid rafts contributes to regulate some biological activities. For example, in basal kerantinocytes, uPAR may sequester the TNF-α converting enzyme (TACE) within lipid rafts, preventing Notch1 activation, and thereby promoting cell proliferation and tumor formation [20]. In endothelial colony-forming cells (ECFCs), inhibition of uPAR expression by antisense oligonucleotides promotes disruption of caveolin-containing lipid rafts, suggesting a prominent role for uPAR in caveolae organization [21]. uPA binding increases uPAR localization to lipid rafts [22], thus probably promoting its binding to VN, which binds preferentially to raft-associated dimeric uPAR [11]. More recently, it has been reported that uPA binding to uPAR induces a raft-localized integrin signaling switch that mediates the hypermotile phenotype of fibrotic fibroblasts [23].

uPAR mediates cell migration towards its specific extracellular ligands; further, the soluble form of uPAR, devoid of the N-terminal DI domain and exposing the chemotactic sequence SRSRY (aa 88-92) binds and activate FPRs, inducing directional cell migration [1,14]. FPRs are involved in uPA-induced cell migration and, in turn, uPAR is involved in FPR-mediated cell migration [1,14]. We have previously demonstrated that uPAR expression was required for migration of PC3 cells towards serum, which contains various and different chemoattractants. We also showed that uPAR-negative HEK-293 cells efficiently migrated towards serum but, after uPAR ectopic expression, they migrated only in a uPAR-dependent manner. In fact, uPAR neutralization, i.e., impairment of uPAR interactions with its signaling partners, or inhibition of signaling mediators activated by uPAR stimulation, impaired directional migration of uPAR-293 cells, even though they were able to migrate in the absence of uPAR expression [9].

These results prompted us to investigate the role of a part of uPAR molecule that we had not neutralized: the GPI-tail. We evaluated its role in migration of PC3 cells, which express high levels of endogenous uPAR, and compared PC3 cell migration mechanisms to migration mechanisms of uPAR-transfected HEK-293 cells, since these cells migrate even in the absence of uPAR expression.

We first demonstrated that uPAR interactions with integrins and FPR1 are required for migration of PC3 cells, as well as active Rho and Rac GTPases, as previously shown for uPAR-293 cells [9]. Then, we showed that degradation of the GPI-tail by PI-PLC impaired PC3 cell migration, and, unexpectedly, did not affect migration of uPAR-293 cells. Indeed, migration of PI-PLC-treated uPAR-293 cells was not blocked by an anti-uPAR antibody, demonstrating that degradation of uPAR-GPI tail restored their capability to migrate in the absence of uPAR expression. A simple explanation of this result may be that PI-PLC induces uPAR release from the cell surface, and thus, PC3 cells, which do not possess an uPAR-independent migration mechanism, remain blocked, whereas uPAR-293 cells behave as uPAR-negative HEK-293 cells and are still able to migrate, using the alternative uPAR-independent migration mechanism. However, HEK-293 cells expressing a transmembrane uPAR migrate using the uPAR-independent mechanisms, as PI-PLC treated uPAR-293 cells, demonstrating that the GPI-tail of uPAR is required for the control of migration mechanisms.

Since the GPI-anchor allows uPAR to associate with lipid rafts, where intracellular signaling mediators are concentrated, we investigated whether lipid rafts may play a role in uPAR-controlled cell migration. In fact, disruption of lipid rafts by MCD in both PC3 and uPAR-293 cells had the same effects on cell migration as the degradation/substitution of the GPI tail of uPAR. Indeed, MCD treatment inhibited migration of PC3 cells and did not impair migration of uPAR-293 cells. However, again, migration of MCD-treated uPAR-293 cells was not impaired by the anti-uPAR antibody, thus suggesting recovery of uPAR-independent migration mechanisms.

These results strongly suggest that the GPI-tail of uPAR is required to control migration mechanisms. In fact, the only way to neutralize uPAR control on uPAR-293 cell migration was to disrupt lipid rafts or to abolish uPAR association with them by GPI-tail hydrolysis or replacement with a transmembrane anchor.

We continued our studies on PC3 cells to explore the relationship between lipid rafts, uPAR signaling partners and migration mechanisms. Cell migration requires the orchestrated regulation of integrin adhesion/deadhesion dynamics and actin cytoskeleton rearrangements [24]. Integrins can exist in several functional states; their activity can be regulated by conformational changes that lead to higher affinity or by lateral association of integrins into clusters on the plasma membrane, which increases ligand binding avidity by providing multiple contact sites [25]. Several observations have led to the idea that inactive integrin is restrained by the cytoskeleton and that integrin ligands induce cytoskeletal release and integrin mobility on the cell membrane and integrin clustering [26]. Integrin clustering at lipid raft domains has been shown to be important for integrin activity [24]. Lipid rafts are strongly enriched in GPI proteins, which have been observed close to integrin nanoclusters, suggesting their involvement in integrin mobility along the cell surface and recruitment in lipid rafts [24]. Consistent with these reports, our results suggest that uPAR association with β1 integrins promotes their partition into lipid rafts. Impairment of this association affects β1 integrins partition to lipid rafts and, presumably, their activity, which is crucial for cell migration.

uPAR can bind simultaneously to both integrins and FPRs, since their binding sites are different [3,27]. FPRs partitioning to lipid rafts has been poorly investigated and results appear to be controversial [28,29]. In PC3 cells, we found that FPR1, which is the most abundantly expressed FPR, as in uPAR-293 cells, in basic conditions associates with lipid rafts. Inhibition of uPAR interaction with FPR1 affected partitioning of FPR1 to lipid rafts and also impaired PC3 cell migration. Interestingly, impairment of the uPAR-FPR1 interaction also affected partitioning of β1 integrins into lipid rafts. By contrast, inhibition of uPAR interaction with β1 integrins did not influence FPR1 localization in lipid rafts, suggesting that uPAR needs to bind FPR1 in order to drive β1 integrins to lipid rafts.

Together, these observations suggest that uPAR binds to and drives integrins to lipid rafts in a highly aggressive cell line such as PC3 cells, thus promoting a potentially crucial event for an efficient integrin activation [24]; however, uPAR may exert this activity only in a FPR-dependent manner. This observation is consistent with previous reports showing that FPR activation, which can be induced by its endogenous ligand uPAR [30], can regulate the activity of various integrins [31,32,33].

In conclusion, we have shown that uPAR controls cell migration mechanisms by connecting FPR1 and integrins and driving this supramolecular complex to lipid rafts for signaling; impairment of these interactions affects integrins partitioning to lipid rafts and, as a consequence, cell migration.
